# Role of HMGB1 in Cisplatin-Persistent Lung Adenocarcinoma Cell Lines

**DOI:** 10.3389/fonc.2021.750677

**Published:** 2021-12-13

**Authors:** Rodolfo L. Chavez-Dominguez, Mario A. Perez-Medina, Jose S. Lopez-Gonzalez, Miriam Galicia-Velasco, Margarita Matias-Florentino, Santiago Avila-Rios, Uriel Rumbo-Nava, Alfonso Salgado-Aguayo, Claudia Gonzalez-Gonzalez, Dolores Aguilar-Cazares

**Affiliations:** ^1^ Laboratorio de Cancer Pulmonar, Departamento de Enfermedades Cronico-Degenerativas, Instituto Nacional de Enfermedades Respiratorias “Ismael Cosio Villegas”, Mexico, Mexico; ^2^ Posgrado en Ciencias Biologicas, Universidad Nacional Autonoma de Mexico, Mexico, Mexico; ^3^ Laboratorio de Quimioterapia Experimental, Escuela Nacional de Ciencias Biologicas, Instituto Politecnico Nacional, Mexico, Mexico; ^4^ Centro de Investigacion en Enfermedades Infecciosas, Instituto Nacional de Enfermedades Respiratorias “Ismael Cosio Villegas”, Mexico, Mexico; ^5^ Clinica de Neumo-Oncologia, Instituto Nacional de Enfermedades Respiratorias “Ismael Cosio Villegas”, Mexico, Mexico; ^6^ Laboratorio de Enfermedades Reumaticas, Departmento de Fibrosis Pulmonar, Instituto Nacional de Enfermedades Respiratorias “Ismael Cosio Villegas”, Mexico, Mexico; ^7^ Facultad de Economia, Universidad Autonoma Benito Juarez de Oaxaca, Oaxaca, Mexico

**Keywords:** lung adenocarcinoma cell lines, non-small-cell lung carcinoma, cisplatin, persistent cells, HMGB1, receptor for advanced glycation end products, immunogenic cell death, overall survival

## Abstract

Significant advances have been made recently in the development of targeted therapy for lung adenocarcinoma. However, platinum-based chemotherapy remains as the cornerstone in the treatment of this neoplasm. This is the treatment option for adenocarcinomas without *EGFR* gain-of-function mutations or tumors that have developed resistance to targeted therapy. The High-Mobility Group Box 1 (HMGB1) is a multifunctional protein involved in intrinsic resistance to cisplatin. HMGB1 is released when cytotoxic agents, such as cisplatin, induce cell death. In the extracellular milieu, HMGB1 acts as adjuvant to induce an antitumor immune response. However, the opposite effect favoring tumor progression has also been reported. In this study, the effects of cisplatin in lung adenocarcinoma cell lines harboring clinically relevant mutations, such as *EGFR* mutations, were studied. Subcellular localization of HMGB1 was detected in the cell lines and in viable cells after a single exposure to cisplatin, which are designated as cisplatin-persistent cells. The mRNA expression of the receptor for advanced glycation end products (RAGE), TLR-2, and TLR-4 receptors was measured in parental cell lines and their persistent variants. Finally, changes in plasma HMGB1 from a cohort of lung adenocarcinoma patients without *EGFR* mutation and treated with cisplatin-based therapy were analyzed. Cisplatin-susceptible lung adenocarcinoma cell lines died by apoptosis or necrosis and released HMGB1. In cisplatin-persistent cells, nuclear relocalization of HMGB1 and overexpression of HMGB1 and RAGE, but not TLR-2 or TLR-4, were observed. In tumor cells, this HMGB1–RAGE interaction may be associated with the development of cisplatin resistance. The results indicate a direct relationship between the plasma levels of HMGB1 and overall survival. In conclusion, HMGB1 may be an effective biomarker associated with increased overall survival of lung adenocarcinoma patients.

## Introduction

Lung cancer is the leading cause of cancer-related deaths worldwide, and non-small cell lung carcinoma (NSCLC) accounts for approximately 85% of diagnosed lung cancers (1). Within this group, lung adenocarcinoma is the most prevalent histological type (1). For lung adenocarcinoma treatment, different antitumor agents are used; however, targeted therapy based on a molecular profile of lung adenocarcinoma is advantageous. In this setting, the administration of tyrosine kinase inhibitors (TKIs) is indicated for patients whose tumors harbor *EGFR* mutations. Because not all patients are candidates for molecular targeted therapy or develop resistance to treatment with TKIs, the remaining option is platinum-based chemotherapy (2, 3).

Cisplatin (CDDP) covalently binds to the N7 position of guanine and adenine residues to form intra- and inter-strand DNA adducts, which disrupts DNA replication. In addition to NSCLC treatment, CDDP is used for the treatment of ovarian, genitourinary, head and neck, and testicular cancers. However, after continuous exposure, tumors develop drug resistance. Many cellular mechanisms implicated in CDDP resistance have been described previously (4, 5).

Considering the heterogeneous nature of tumors, some tumor cells may exhibit intrinsic resistance during the initial cycles of treatment. These cells can either exhibit low drug sensitivity (tolerant cells), allowing them to grow in the presence of drug, or they may be arrested and enter a dormant state (persistent cells) to escape therapeutic drug-induced cell death. Either of these events can lead to subsequent treatment failure and tumor relapse (6).

Previous reports have suggested that High-Mobility Group Box 1 (HMGB1) is involved in drug resistance in several cancers. HMGB1 is a nuclear protein that participates in DNA repair, including nucleotide excision repair, base excision repair, mismatch repair, and double-strand break repair *via* non-homologous end-joining (NHEJ). This suggests that HMGB1 may contribute to DNA repair-related radioresistance and chemoresistance (7, 8).

HMGB1 is an essential activator of the early cellular response to genotoxic insults induced by platinum drugs because of its strong binding affinity for CDDP-modified DNA. *In vitro* studies demonstrated that HMGB1 is implicated in NHEJ by stimulating ligation even without compatible DNA ends (9, 10). NHEJ has been correlated with chemoresistance and radioresistance in a variety of cancer types, including colorectal, pancreatic, glioma, and hepatocellular carcinoma (11–14).

In some cancers, HMGB1 inhibits apoptosis by triggering autophagy (15–17) and is considered an important regulator of autophagy (18, 19). In addition, HMGB1 has been reported to participate in tumor progression, invasion, and metastasis. In some cancers, the increased expression of HMGB1, detected as a transcript or protein, has been associated with tumor progression. Several studies have reported the overexpression and release of HMGB1 in breast (20), gastric (21, 22), hepatocellular (23, 24), colorectal (25), and pancreatic (26) cancers and NSCLC (27–30).

There is evidence to suggest that dying or stressed cells release specific intracellular molecules that act as internal alarm signals. These signals, known as damage-associated molecular patterns (DAMPs), function as adjuvant molecules to trigger the immune response. In the tumor microenvironment, these DAMPs, particularly HMGB1, trigger an antitumor immune response. HMGB1, acting as a “find me” signal, activates cells through Toll-Like Receptor (TLR) 2 and 4, as well as the receptor for advanced glycation end products (RAGE). These receptors activate the NF-κB signaling pathway which triggers an inflammation process that release pro-inflammatory cytokines. Some reports indicate that the chemotherapeutic drugs oxaliplatin and anthracycline induce a type of cell death known as immunogenic cell death (ICD) in colon cancer (31). Markers of ICD include membrane exposure of calreticulin and the release of ATP and HMGB1, among others (32). When ICD is induced, HMGB1 is released and interacts with TLR-2 and TLR-4 to activate the host immune response. Some studies also indicate that CDDP promotes the release of DAMPs independently of ICD induction (33, 34).

Owing to the participation of HMGB1 in intrinsic CDDP resistance and also as a DAMP released during CDDP-induced cell death, we investigated the biological role of HMGB1 in the response to chemotherapy. In this study, the effects of CDDP in lung adenocarcinoma cell lines containing different clinically relevant mutations, including *EGFR* mutations, were examined. Subcellular localization of HMGB1 was also detected in cell lines and in CDDP-persistent cells, which remain viable after cytotoxic drug exposure. Changes in mRNA expression of the RAGE, TLR-2, and TLR-4 receptors were analyzed both in parental cell lines and their persistent variants. Finally, changes in plasma HMGB1 levels in a cohort of lung adenocarcinoma patients without *EGFR* mutations and treated with CDDP-based chemotherapy were determined.

## Materials and Methods

### Lung Adenocarcinoma Cell Lines

The lung adenocarcinoma cell lines HCC827 (E746-A750) and HCC4006 (L747-E749), harboring an *EGFR* exon 19 deletion, and the A549 and SKLU-1 cell lines were obtained from the ATCC (Manassas, VA, USA). The 1.3.11 cell line, established by our group from the pleural effusion of a treatment-free patient with lung adenocarcinoma, was also included (35, 36). The cell lines were cultured in RPMI-1640 medium supplemented with 10% heat-inactivated fetal bovine serum and 1% antibiotics and incubated in a humidified atmosphere at 37°C with 5% CO_2_. After reaching confluence, adherent cells were harvested by enzymatic treatment.

### Next-Generation Sequencing Mutational Status of Lung Adenocarcinoma Cell Lines

To identify mutations in lung adenocarcinoma cell lines, we employed targeted sequencing using the TruSight tumor 15 kit (Illumina, San Diego, CA, USA). In brief, total DNA from confluent cell cultures was extracted using the Pure link genomic DNA mini kit (Thermo Fisher, Waltham, MA, USA). The purity of DNA exceeded 1.8, based on the 260/280 absorbance ratio, and quantification was done using a Qubit 4 fluorometer (Thermo Fisher, Waltham, MA, USA). A DNA library was prepared following the instructions of the manufacturer and sequenced on a MiSeq Illumina device (Illumina, San Diego, CA, USA) to analyze mutations in 15 candidate driver genes (*AKT1*, *BRAF*, *EGFR*, *ERBB2*, *FOXL2*, *GNA11*, *GNAQ*, *KIT*, *KRAS*, *MET*, *NRAS*, *PDGFRA*, *PIK3CA*, *RET*, and *TP53*) associated with solid tumors.

### Dose–Response Curves of CDDP in Lung Adenocarcinoma Cell Lines

To measure CDDP cytotoxicity, we used the 3-(4,5-dimethylthiazol-2-yl)-2,5-diphenyltetrazolium bromide (MTT) assay (Trevigen, Gaithersburg, MD, USA). Briefly, 1.2–3 × 10^4^ cells from the A549, 1.3.11, HCC4006, HCC827, and SKLU-1 cell lines were seeded into 96-well plates and cultured overnight. The cells were treated with serial dilutions of CDDP ranging from 160 to 5 μM for 24 h. The concentration range represented the reported plasma concentrations detected in treated patients with this cytotoxic drug (37). After exposure, 10 μL of MTT were added to each well, and the plate was incubated at 37°C for 4 h. Formazan crystals were dissolved by adding 150 μL of dimethyl sulfoxide, and absorbance was measured at 560 nm using a Multiskan Ascent plate reader (Thermo, Waltham, MA, USA). Cell viability was calculated with respect to untreated cells which represented 100% viability. The half-maximal inhibitory concentration was calculated. For subsequent experiments, the adenocarcinoma cell lines were exposed to CDDP at concentrations inducing the highest cytotoxicity over the treatment period.

### Percentage of Viable, Apoptotic, and Necrotic Cells

Lung adenocarcinoma cell lines were cultured in T-25 flasks in the presence of CDDP, and after treatment, the cells were collected and split into two fractions. In one fraction, we quantified the percentages of viable, apoptotic, and necrotic cells. The other fraction was used to measure caspase-3/7 activity (see below). Briefly, the cells were washed with ice-cold Ca^2+^Mg^2+^-free phosphate-buffered saline (PBS) and rinsed in binding buffer, and 2–3 × 10^5^ cells were stained with FITC Annexin-V/PI following the instructions of the FITC Annexin V Apoptosis Detection kit II (BD Biosciences, San Diego, CA, USA). As apoptosis and necrosis are time-dependent processes, the measurement was conducted at different times. A total of 15,000 events were immediately acquired using a FACS Canto II flow cytometer (Becton Dickinson, Franklin Lakes, NJ, USA). Two independent experiments were done. The percentage of viable, apoptotic, and necrotic cells was obtained using FlowJo V10 software (FlowJo, Ashland, OR, USA).

### Caspase-3/7 Activity Assay

To determine whether apoptosis was mediated by the activation of effector caspases, we measured caspase-3/7 activity in the remaining cell fractions derived from the previous apoptotic/necrotic assay using the caspase-3/7 fluorometric assay kit (R&D systems, Minneapolis, MN, USA). Briefly, the collected cells were centrifuged, and the pellets were rinsed in ice-cold lysis buffer and incubated on ice for 10 min. The cell extracts were centrifuged at 10,000 × *g*, and 50 μL of each extract was mixed with 50 μL of reaction buffer containing the DEVD-AFC substrate in a black 96-well plate. For each cell line, positive (extract of apoptotic cells), negative (cell extract obtained from non-treated cells), and blank controls were included according to the instructions of the manufacturer. The plate was incubated at 37°C in the dark for 2 h, and fluorescence was measured using the Fluoroskan Ascent FL microplate reader (Thermo, Waltham, MA, USA) at 390- and 485-nm excitation and emission, respectively. In addition, protein was quantified using the Micro BCA protein assay kit (Pierce, Waltham, MA, USA) with the Multiskan Ascent plate reader (Thermo, Waltham, MA, USA) at 562 nm. Caspase-3/7 activity (relative fluorescence units) per milligram of protein was calculated, and the data are presented as fold-change relative to CDDP-treated cells from each cell line to their respective untreated cells.

### LDH Assay

To confirm the cytotoxic effect induced by CDDP, the release of cytosolic enzyme lactate dehydrogenase (LDH) was measured in the cell culture supernatants using the cytotoxicity detection kit (Roche Diagnostics, Germany). Cells were seeded into 96-well plates under the same experimental conditions previously mentioned. After treatment, the supernatants were collected and centrifuged, and 50 μL were transferred into a plate and mixed with 150 μL of working reaction mixture. Negative (cells without treatment) and positive (cells treated with 1% Triton X-100) controls were included according to the instructions of the manufacturer. The plate was incubated in the dark at room temperature (RT) for 30 min. Absorbance was measured at 490 nm using the Multiskan Ascent plate reader. The percentage of LDH release was calculated according to the equation previously reported (38).

### Calreticulin Exposure Detected by Flow Cytometry

Previous studies have demonstrated that calreticulin exposure on cell membranes is an early event in the ICD process (31, 39). For this reason, calreticulin exposure was measured at times preceding the emergence of morphological and biochemical changes associated with apoptotic cell death. After treatment, cells were collected and washed twice in cold PBS containing 1% albumin and 0.1% sodium azide. The cells were then incubated with a PE-conjugated anti-calreticulin antibody for 30 min at 4°C (Abcam, Cambridge, MA, USA). After incubation, the cells were washed with cold PBS and incubated with 7-AAD solution for 10 min. Finally, at least 15,000 events were acquired using the FACS Canto II flow cytometer. Data was analyzed using FlowJo V10 software gating the population of viable (7-AAD negative) cells. The mean fluorescence intensity is presented.

### Detection of Membrane Calreticulin Using Confocal Microscopy

To confirm the results obtained by flow cytometry, calreticulin exposure was assessed using confocal microscopy in the A549 cell line. After cell culture in 35-mm petri dishes, CDDP was added at different times. The cells were washed and fixed in 4% paraformaldehyde in PBS for 10 min. Blocking solution was added to avoid non-specific binding, and the cells were incubated with Alexa-488-conjugated anti-calreticulin primary antibody (Bioss, Woburn, MA, USA) for 1 h. After washing, the cell membrane was stained using Alexa-633-conjugated wheat germ agglutinin and with 4′,6-diamidino-2-phenylindole (DAPI) for nuclear staining. Finally, the cells were visualized using an Olympus FV1000 (Olympus Life Science, Center Valley, PA, USA) confocal microscope, and representative images were acquired.

### ADP/ATP Ratio

ADP/ATP ratios were measured at time points in which the maximum percentage of apoptotic cells and highest activity of caspase-3/7 were detected. The ApoSENSOR ADP/ATP Ratio Assay Kit (Enzo Life Sciences, Farmingdale, NY, USA) was used according to the directions of the manufacturer. Supernatants from cells cultured in 96-well plates were collected for subsequent HMGB1 measurement (see below), whereas the adherent cells were treated with 60 μL of nucleotide-releasing buffer, and the plate was incubated at RT for 5 min. To quantify intracellular ATP, 100 μL of ATP monitoring enzyme was mixed with 50 μL of cell extract in a 96-well plate. The bioluminescent signal was recorded immediately using a Fluoroskan Ascent (Thermo, Waltham, MA, USA) luminometer. After adding 10 μL of ADP-converting enzyme, intracellular ADP was measured. The ADP/ATP values are presented.

### HMGB1 Released From Lung Adenocarcinoma Cell Lines

In supernatants collected from the ADP/ATP assay, extracellular HMGB1 was measured using an enzyme-linked immunosorbent assay (IBL International GMHB, Hamburg Germany) following the instructions of the manufacturer. Optical density was measured at 450 nm using a Multiskan Ascent plate reader.

### HMGB1 Localization in CDDP-Persistent Adenocarcinoma Cells

Intracellular HMGB1 in persistent cells was detected using indirect immunofluorescence staining. Lung adenocarcinoma cell lines cultured in eight-chamber slides were washed, fixed with ethanol, and permeabilized with PBS containing 1% sodium dodecyl sulfate for 5 min. After washing, the cells were treated for 30 min with blocking solution to avoid non-specific binding. Then, the slides were incubated overnight in a humified chamber at 4°C with polyclonal anti-HMGB1 human antibody (1:100) acquired from Abcam, Cambridge, UK. After washing, the slides were incubated with an Alexa Fluor 488-conjugated anti-rabbit secondary antibody (1:250) from Molecular Probes, CA, USA, at 32°C for 90 min. The cells were then incubated with DAPI (Sigma-Aldrich, St. Louis, MO, USA) for 15 min for nuclear staining. Finally, the slides were mounted with Vectashield (Vector Laboratories, CA, USA), and the cells were observed using an epifluorescence microscope from Leica Microsystems (Wetzlar, Germany).

HMGB1 localization in tumor cells was classified as follows: nuclear, cytoplasmic, or nuclear/cytoplasmic. Digital images of high-power fields (HPFs) were acquired. The HPFs were captured using a DFC425 C color camera coupled to a Leica microscope and evaluated by two independent pathologists using Leica Application Systems V.3.6.0 software (Leica Microsystems, Inc.).

### Expression of HMGB1, RAGE, TLR-2, and TLR-4 in CDDP-Persistent Cells

To evaluate changes of *HMGB1*, *TLR-2*, *TLR-4*, and *RAGE* at the mRNA level, qPCR analysis was performed in lung adenocarcinoma and persistent cells. Briefly, cell lines cultured in T-25 flasks were exposed to CDDP for 24 h, and after washing to eliminate dying and dead cells, the adherent cells were collected. Total RNA was isolated using the PureLink RNA Minikit (Ambion, Austin, TX, USA). cDNA was synthesized using the High Capacity cDNA Reverse Transcription Kit (Ambion, Austin, TX, USA). Taqman probes were used to amplify *HMGB1*, *TLR-2*, *TLR-4*, and *RAGE* mRNA. β-Actin was used as an endogenous control. Gene expression was detected using the 7500 Real Time PCR System (Applied Biosystems, Foster City, CA, USA). Data were normalized using the expression of the housekeeping gene, β-Actin, and relative expression was calculated by the ΔΔCT method.

### Western Blotting

In order to validate the previous RT-qPCR results, the protein expression level of RAGE was verified by Western blot. Cells were cultured in T-75 flasks and treated under the same experimental conditions mentioned above. As a positive control of constitutive RAGE expression, THP-1 monocytic cell line (ATCC, Manassas, VA, USA) was used (**Supplementary Figure S1**). Following treatment, cytoplasmic and cell membrane extracts were obtained using the Subcellular Protein Fractionation Kit for Cultured Cells (Thermo Scientific, Waltham, MA, USA). Protein was quantified using the MicroBCA Protein Assay Kit (Pierce, Waltham, MA, USA). A total of 30 mg of protein per lane was resolved in 10% SDS-PAGE and subsequently transferred onto a nitrocellulose membrane. After 1 h of blockade of non-specific sites with 2% of Bovine Serum Albumin in PBS, membranes were incubated with primary antibodies against RAGE (Abcam, Cambridge, MA, USA) or β-Actin (Sigma, Burlington, MA, USA) (1:200) at 4°C overnight. After washing, the membranes were incubated with biotinylated anti-rabbit (Dako, Santa Clara, CA, USA) or anti-mouse (GeneTex, Alton Pkwy Irvine, CA, USA) secondary antibodies (1:300) for 1 h at RT. Protein bands were visualized by employing the Vectastain Elite ABC peroxidase kit (Vector Laboratories, Burlingame, CA, USA) and DAB-H_2_O_2_ system (Sigma, Burlington, MA, USA).

### HMGB1 Quantification in a Cohort of Lung Adenocarcinoma Patients Treated With CDDP Study Population

From August 2013 to December 2018, patients attending the Pneumo-oncology Department of the Instituto Nacional de Enfermedades Respiratorias Ismael Cosio Villegas were recruited. The inclusion criteria were as follows: patients with a confirmed diagnosis of primary pulmonary adenocarcinoma according to WHO criteria and by histological examination of biopsy specimens or cytological observation of malignant cells in pleural effusion. The cytological or histopathological diagnoses were made by two pathologists. Only patients whose biopsies did not contain any *EGFR* mutations, as detected by the Idylla *EGFR* mutation assay from Biocartis (NV, Mechelen, Belgium), were included. All procedures were routinely done in the pathology department. Based on the TMN score, all patients were diagnosed with clinical stage IIIB and IV disease.

In addition, only patients with a Karnofsky-based performance status of 80–100%, complete clinical and laboratory data, and clinical follow-up were included. During follow-up, the patients had acceptable hematologic, hepatic, and renal function. Patients with comorbidities, such as severe cardiopulmonary dysfunction, uncontrolled arrhythmia, myocardial ischemia history, or active infection, were excluded. Patients who presented with CDDP toxicity, hepatic or renal failure, or infection were also excluded.

A total of 100 lung adenocarcinoma patients were enrolled in this study. The overall survival (OS) of patients was designated as the time elapsed from the initiation of first-line chemotherapy to the date of death. The average median OS for the entire group was obtained from a Kaplan–Meier curve. Based on this data, the patients were grouped according to shorter (≤ 12 months) or longer (≥ 12 months) OS.

For controls, 26 healthy non-smokers and 29 heavy smokers were included. The subjects from the control groups had normal values for lung function as measured by spirometry. The Committee of Science and Bioethics of the Instituto Nacional de Enfermedades Respiratorias approved the protocol for the collection of biological samples. The patients were informed of the protocol before approving and signing the informed consent for blood collection during chemotherapy. Written informed consent was obtained from all participants. The demographic characteristics of the patient and control groups are presented in **Tables 1**, **2**.

**Table 1:** Demographic data and clinical characteristics of the cohort of patients with lung adenocarcinoma.

Characteristics	Total group	Patients with		p
		Shorter survival^a^	Longer survival^a^	
*n*	100	41	59	–
Age, years	59 (25–83)^b^	57 (25–76)^b^	61 (27–83)^b^	0.04
Female	45	16	29	–
Male	55	25	30	–
Stage				
IIIb/IV	34/66	15/26	19/40	ns
Karnofsky				
80/90/100	67/22/11	28/10/3	39/12/8	ns
Treatment (first-line regimen)				
CDDP/paclitaxel	51	23	28	–
CDDP/pemetrexed	22	8	14	–
CDDP/vinorelbine	20	7	13	–
CDDP/gemcitabine	7	3	4	–
Median OS (months)	12	9	21	0.0001
HMGB1 (ng/mL) t0	1.7 (0.2–8.6)^b^	1.6 (0.2–4.7)^b^	1.7 (0.4–8.6)^b^	ns
HMGB1 (ng/mL) t1	1.8 (0.4–4.6)^b^	1.8 (0.5–4.3)^b^	1.8 (0.4–4.6)^b^	ns
HMGB1 (ng/mL) t2	2.0 (0.5–7.9)^b^	1.9 (0.5–7.4)^b^	2.1 (0.5–6.0)^b^	ns

a Patients were categorized according to median overall survival.

b Median (min–max).

ns, statistically non-significant; t0, before the first cycle of chemotherapy; t1, before the third cycle of chemotherapy; t2, before the sixth cycle of chemotherapy.

**Table 2:** Demographic characteristics of control groups.

	Total	Non-smokers	Smokers
*n*	55	26	29
Age, years		56 (43–83)^a^	52 (45–63)^a^
Gender			
Female	30	15	15
Male	25	11	14
HMGB1 (ng/mL)		1.9 (0.9–2.8)^a^	2.5 (0.9–4.6)^a^

a Median (min–max).

### Blood Sample Collection

Blood samples were collected from patients longitudinally during the follow-up and just before the first (t0), third (t1), and sixth cycle (t2) of chemotherapy. For all participants, blood samples (9–12 mL) were collected in EDTA-vacutainer tubes and centrifuged at 1,500 rpm, and the resulting plasma was immediately stored at −80°C until the HMGB1 ELISA assay was performed.

### Quantification of HMGB1 in Plasma Samples From Lung Adenocarcinoma Patients

The plasma samples collected during treatment were thawed and immediately processed. All plasma samples were assayed in duplicate, and the three collected samples from each patient were quantified using the same ELISA plate to avoid inter-assay variation. The concentration of plasma HMGB1 was determined using the ELISA assay employed for HMGB1 release from the lung adenocarcinoma cell lines described above.

### HMGB1 Expression and Association With OS in the LUAD-TCGA Cohort

The clinical impact of HMGB1 in CDDP-treated patients was determined by using datasets from the Lung Adenocarcinoma project of The Cancer Genome Atlas (LUAD-TCGA). The data were downloaded using the R (V.4.0.4) packages RTCGA (V.1.20) and TCGAbiolinks (V.2.18). Data was curated to eliminate duplicate samples, and only data from patients treated with CDDP at an advanced stage with OS and HMGB1 expression data were analyzed. Using the maximally selected rank statistics method (40), the samples were categorized according to HMGB1 expression. OS analysis was performed using GraphPad Prism V.8 software.

### Relationship of HMGB1 Plasma Concentration With OS in the Cohort of Lung Adenocarcinoma Patients

The plasma fluctuations of HMGB1 during CDDP treatment were quantified and associated with OS from the patients. Patients with lung adenocarcinoma were followed up by the pneumologist and oncologist from the time of diagnosis during first-line chemotherapy through subsequent treatments. Clinical data including survival were registered in the medical record, and these data were used to build a database.

### Statistical Analysis

The cell line data was expressed as mean ± standard deviation (SD). Data obtained from distinct experimental conditions were compared using a paired Student’s *t*-test. For lung adenocarcinoma patients, the data are presented as median ± interquartile range. The non-parametric Kruskal–Wallis test and Dunn’s multiple-comparisons test were used to compare the plasma concentrations of HMGB1 from the patient cohort. Receiver operating characteristic (ROC) curves were constructed to determine the cutoff value of plasma HMGB1 concentration. HMGB1 plasma values from smoker subjects were compared to those values from patients with adenocarcinoma at t0. Using a cutoff value for plasma HMGB1 concentration, OS curves were generated using the Kaplan–Meier method, and the differences between the curves were analyzed using the Wilcoxon test. Finally, the relationship between HMGB1 levels and the mean OS of patients was analyzed using the extended Cox model. This model estimates the observations coming from the same patient over time based on serial correlations between observations. An important assumption is that the presence of an event in an individual is not independent of a previous event, so the model shows the estimate with a correction for this dependence. The model was calculated using the Huber–Sandwich estimator. ANOVA, graphs, and Kaplan–Meier analysis were done using GraphPad Prism V.8 software. The extended Cox model was performed using the R language V.4.0.4. Values of *p <*0.05 were considered statistically significant.

## Results

### Lung Adenocarcinoma Cell Lines

#### Mutational Status and Cytotoxicity of CDDP-Treated Lung Adenocarcinoma Cell Lines

**Figure 1:**
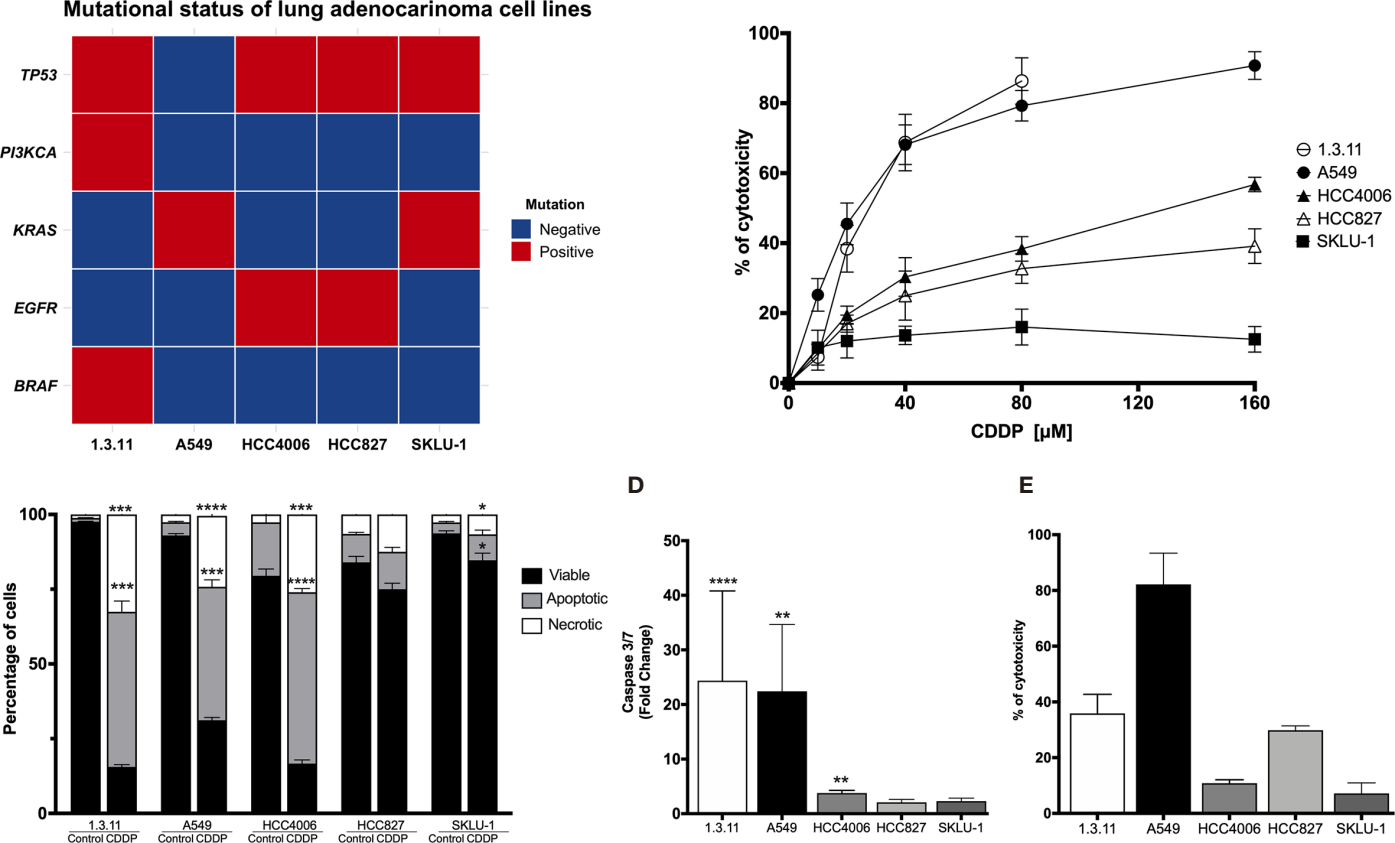
Cell death induction in lung adenocarcinoma cell lines by cisplatin (CDDP). **(A)** Heat map showing clinical mutations in lung adenocarcinoma cell lines using TruSight tumor 15. Two experiments were done. **(B)** Dose–response curves of lung adenocarcinoma cell lines exposed to CDDP for 24 h. For detection, an MTT assay was used. Three experiments were done in triplicate. **(C)** Percentages of viable, apoptotic, and necrotic cells are shown. Lung adenocarcinoma cell lines were treated with the CDDP concentration that induced the highest level of apoptotic cell death. A comparison of the distinct proportions of viable, apoptotic, and necrotic cells in untreated (control) *versus* CDDP-treated cells is shown. Three independent experiments were done in triplicate. **(D)** Caspase-3/7 activity. This parameter was directly correlated with the proportion of apoptotic cells detected previously. Fold-change with respect to the fluorescence units of CDDP-treated cells compared with that of untreated cells are shown. **(E)** Percentage of cytotoxicity measured by lactate dehydrogenase (LDH) enzyme activity. Supernatants from cell culture were collected following 24 h of CDDP treatment. The percentage of released LDH is indicated. Three experiments were done in triplicate. All data are shown as mean ± SD. The significant difference between treatment and control is indicated with asterisks (*p < 0.05, **p < 0.005, ***p < 0.0005, ****p < 0.0001).

A next-generation sequencing analysis of 15 cancer driver genes associated with solid tumors was performed. The data obtained confirmed the presence of the characteristic *EGFR* mutation in the lung adenocarcinoma cell lines HCC4006 and HCC827. Other clinically relevant mutations, such as *TP53* and *KRAS*, were detected in most cell lines. In particular, the 1.3.11 cell line harbors *BRAF* and *PIK3CA* mutations (**Figure 1A**).

For A549, the highest CDDP concentration resulted in approximately 90% cytotoxicity. A similar percentage of cytotoxicity was found in the 1.3.11 cell line; however, this effect required only half the concentration of CDDP. The HCC827 and HCC4006 cell lines exhibited mild sensitivity and reached 40–60% of cytotoxicity at the highest CDDP concentration. In contrast, the SKLU-1 cell line exhibited CDDP resistance as less than 20% cell death was observed (**Figure 1B**). In general, CDDP-sensitive cells displayed apoptotic or necrotic features. Overall, lung adenocarcinoma cell lines showed different degrees of sensitivity to CDDP, and this phenomenon was independent of the mutational profile.

#### Apoptosis/Necrosis Assessment

To determine whether CDDP-induced cell death was mediated by apoptosis or necrosis, the percentage of dead and dying cells was assessed using the Annexin-V/propidium iodide assay. For the 1.3.11, A549, and HCC4006 cell lines, CDDP induced a similar proportion of apoptotic and necrotic cells. This was detected at different drug exposure times. In the 1.3.11 and A549 cell lines, cells showing apoptotic morphology were detected at early times following drug treatment, and this phenomenon evolved to secondary necrosis. After exposure, HCC4006 showed primarily a necrosis-like morphology. In contrast, HCC827 and SKLU-1 cells were unaffected as they maintained a similar proportion of viable cells with minimal damage induced by CDDP (**Figure 1C**). These results are similar to that observed in cytotoxicity tests performed with MTT.

#### Caspase-3/7 Activity Quantification

To corroborate apoptosis induced by CDDP, the activity of effector caspases 3/7 was measured at time points in which the highest proportion of apoptotic cells was detected. The caspase-3/7 assays confirmed the MTT assay results. The activity of these caspases in A549 and 1.3.11 cells, which exhibited the highest CDDP sensitivity, was increased approximately 25-fold compared with their respective untreated cells. However, the HCC4006, HCC827, and SKLU-1 cell lines showed only a slight increase in the activity of these caspases (**Figure 1D**).

#### LDH Quantification

Cytotoxicity induced by CDDP treatment in the cultured cell lines was detected at the final time point to confirm the cell death process. The A549 cell line, highly sensitive to CDDP, released the highest amount of LDH. The 1.3.11 cell line released approximately 40% LDH. Surprisingly, the HCC4006 cell line, in which CDDP induced a high proportion of necrotic cells and apoptotic bodies, did not show a release of LDH. In HCC827 and SKLU-1, LDH release was slightly increased following CDDP treatment (**Figure 1E**). These results confirm our initial observations from the CDDP cytotoxicity curves. Cell lines A549 and 1.3.11 exhibited the highest LDH release and the highest sensitivity to CDDP. HCC4006, HCC827, and SKLU-1 showed higher resistance to CDDP and lower LDH release. Taken together, these results show that the lung adenocarcinoma cell lines exhibit different degrees of susceptibility to CDDP regardless of mutational profile.

#### ICD Markers in CDDP-Sensitive Cells

**Figure 2:**
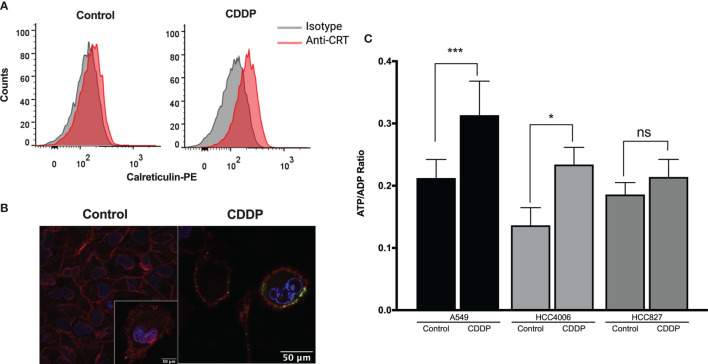
Detection of immunogenic cell death-associated markers in cell lines treated with cisplatin (CDDP). **(A)** Calreticulin was detected on the cell membrane of A549 viable cells using flow cytometry. Representative histograms of control and CDDP-treated cells are shown. **(B)** Images of confocal microscopy showing calreticulin distribution in control and CDDP-treated cells. Magnification: control, ×400; inset and CDDP, ×600. **(C)** ADP/ATP ratio obtained when the highest proportion of apoptotic cells was detected. Three experiments were done in triplicate. Data is expressed as mean ± SD. The significant difference between treatment and control is indicated with asterisks (*p < 0.05, ***p < 0.0005, ns, non-significant).

The presence of some ICD markers in CDDP-treated cells, such as the release of intracellular ATP and HMGB1 as well as the appearance of calreticulin on the cell membrane, was examined. We found that, after 6 h of CDDP exposure, calreticulin was translocated to the cell membrane in approximately 25% of A549 cells (**Figure 2A**). For a more rigorous observation, confocal microscopy was used, and calreticulin was detected as dots colocalized on the cell membrane (**Figure 2B**). In contrast, CDDP did not cause calreticulin translocation to the membrane in HCC4006 and HCC827 cells at time points previously determined to induce apoptotic features.

We also found that, in the A549 and HCC4006 cell lines, CDDP induced a significant increase in the ADP/ATP ratio (*p* = 0.0002 and *p* = 0.01, respectively). This result was caused by a significant increase of ADP and a concomitant decrease of ATP (**Figure 2C**). No significant increase was detected in the HCC827 cell line after CDDP exposure (**Figure 2C**).

Finally, the release of HMGB1 was measured following 24 h of CDDP treatment. We found that CDDP significantly increased the release of HMGB1 in the 1.3.11 (*p* = 0.0002), A549 (*p* = 0.002), and HCC827 (*p* = 0.02) cell lines (**Table 3**). In contrast, CDDP did not alter the release of HMGB1 in the HCC4006 and SKLU-1 cell lines (**Table 3**). In summary, our results suggest that, in all cell lines, only A549 expressed ICD-associated markers during the spatial–temporal cell death process.

**Table 3:** Concentration of HMGB1 released in the supernatants of cisplatin (CDDP)-treated cell lines.

Cell line	HMGB1 concentration			
	Control (ng/mL)	CDDP (ng/mL)	Fold-change	*p*-value
1.3.11	3.95 ± 0.04	6.82 ± 0.02	1.73	0.0002
A549	0.75 ± 0.65	18.22 ± 0.7	24.29	0.0002
HCC4006	7.25 ± 0.57	7.10 ± 0.51	-0.98	0.002
HCC827	2.75 ± 0.21	6.61± 0.19	2.41	0.02
SKLU-1	6.98 ± 0.57	5.85 ± 0.13	-0.84	ns

ns, non-significant.

#### Localization of HMGB1 in Lung Adenocarcinoma Cell Lines and Persistent Cells

**Figure 3:**
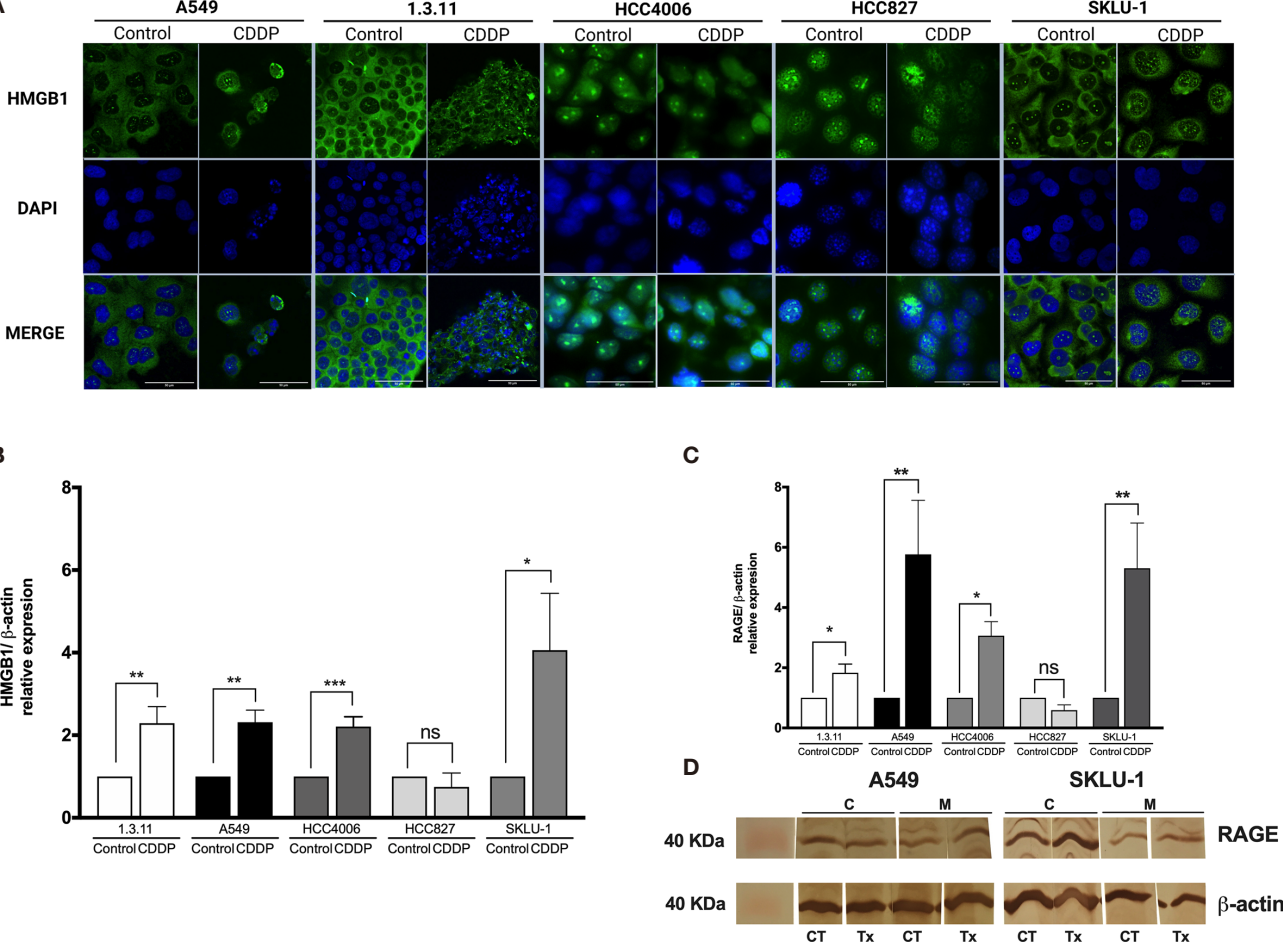
HMGB1 and receptor for advanced glycation end products (RAGE) in lung adenocarcinoma cell lines and in cisplatin (CDDP)-persistent cells. **(A)** HMGB1 is localized to the cytoplasm of untreated tumor cells, and nuclear relocalization occurs after a single exposure to CDDP. For nuclear staining, DAPI was used. Merge is indicated. Indirect immunofluorescence staining was employed. Magnification: ×400. **(B)** HMGB1 and **(C)** RAGE expression was measured by qPCR in untreated tumor cell lines and in CDDP-persistent cells. Three experiments in duplicate were done. The relative expression of each cell line between treated and untreated cells is indicated. **(D)** Protein expression level of RAGE (43 kDa) in CDDP-persistent and untreated cells from A549 and SKLU-1 cell lines. Protein bands detected at around 40 kDa from cytoplasmic (C) or membrane (M) extracts are shown from control (CT) or CDDP (Tx)-treated cells. β-Actin (42 kDa) was employed as a constitutive expression control. Data is shown as mean ± SD. The significant difference between treatment and control is indicated with asterisks (*p < 0.05, **p < 0.005, ***p < 0.0005, ns, non-significant).

In A549 cells, nuclear–cytoplasmic distribution of HMGB1 prior to treatment was observed. In contrast, SKLU-1 and 1.3.11 untreated cells exhibited a cytoplasmic distribution of HMGB1. The HCC4006 and HCC827 cells showed that HMGB1 mainly localized to the nuclei (**Figure 3A**). After CDDP exposure, A549 cells had decreased cytoplasmic staining of HMGB1, and a strong nuclear localization was observed. In the 1.3.11 and SKLU-1 cell lines, CDDP treatment did not alter the cytoplasmic distribution of HMGB1 in the residual viable cells. In the HCC4006 cell line, a strong nuclear localization of HMGB1 was observed in CDDP-persistent cells. In HCC827 cells, CDDP exposure induced the relocalization of HMGB1 from the nuclei to the cytoplasm (**Figure 3A**). These results suggest that the increase of HMGB1 found in cell culture supernatants occurs in dying cells. Nucleocytoplasmic shuttling of HMGB1 may contribute to the repair of DNA lesions and in the development of CDDP resistance.

#### HMGB1, TLR-4, TLR-2, and RAGE Expression

To analyze whether CDDP alters the expression of *HMGB1* mRNA, qPCR was performed. We found a significant increase in *HMGB1* expression in 1.3.11 (*p* = 0.005), A549 (*p* = 0.001), HCC4006 (*p* < 0.001), and SKLU-1 (*p* = 0.02) cells following CDDP treatment; however, no changes were observed in HCC827 cells (**Figure 3B**).

Changes in the expression of the cognate receptors of HMGB1, including *TLR-4, TLR-2*, and *RAGE*, in CDDP-persistent cells were analyzed. With respect to *TLR-4* and *TLR-2* expression, no significant differences were observed between CDDP-persistent and untreated cells (**Table 4**). With respect to *RAGE* expression, a significant increase in 1.3.11, A549, HCC4006, and SKLU-1 was observed after treatment (**Figures 3C, D**). This increase was the highest only in A549 and SKLU-1 cell lines at the mRNA and protein levels (**Figures 3C, D** and **Supplementary Figure S1**). Overall, these results suggest that the HMGB1–*RAGE* axis plays an important role in CDDP resistance following treatment of lung adenocarcinoma cell lines.

**Table 4:** TLR-2 and TLR-4 mRNA expression from cultured cell lines.

Cell line	Relative expression					
	TLR-2/ß-Actin			TLR-4/ß-Actin		
	Control	CDDP	*p*-value	Control	CDDP	*p*-value
1.3.11	1.00	0.12	ns	1.00	1.75	ns
A549	ND	ND	–	1.00	1.00	ns
HCC4006	1.00	0.67	ns	1.00	1.16	ns
HCC827	1.00	ND	–	ND	ND	–
SKLU-1	1.00	ND	–	ND	ND	–

ND, non-detected; ns, non-significant.

### Plasma HMGB1 Concentration in a Cohort of Lung Adenocarcinoma Patients

#### Plasma HMGB1 Concentration in Lung Cancer and Control Groups

**Figure 4:**
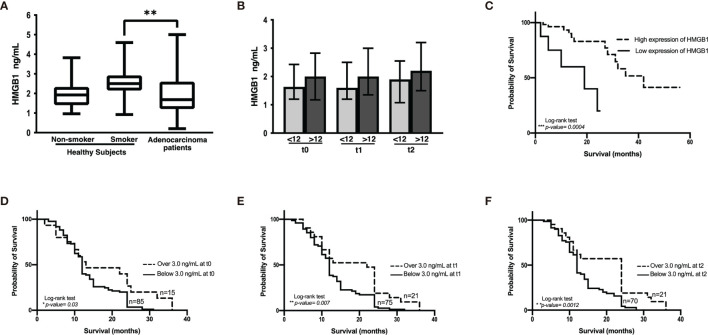
HMGB1 plasma concentration. **(A)** Comparison among healthy non-smokers, smokers, and lung adenocarcinoma patients before the start of therapy. Statistical significance was found in the control smoker group and lung adenocarcinoma patients (**p = 0.0096). **(B)** HMGB1 concentration in the cohort of patients with lung adenocarcinoma classified with respect to shorter (<12 months) and longer (>12 months) overall survival and before the start of treatment (t0) and prior to the third (t1) and to the sixth (t2) chemotherapy cycle. **(C)** Kaplan–Meier analysis of patients from the Lung Adenocarcinoma project of The Cancer Genome Atlas based on HMGB1 expression. **(D–F)**, Kaplan–Meier curves of the cohort of lung adenocarcinoma patients according to HMGB1 plasma concentration at distinct chemotherapy cycles using a cutoff value of 3 ng/mL.

Several reports indicate that smoking induces the lung inflammatory process. As HMGB1 is an important molecule in inflammation, we evaluated HMGB1 expression in healthy non-smokers and smokers. Although no significant differences were detected between non-smokers and smokers, a tendency of increased plasma concentrations of HMGB1 was observed in the smoker group (**Figure 4A**). This suggests the presence of persistent inflammation resulting from cigarette smoke. Because smoking is a risk factor for lung cancer development and a high proportion of lung cancer patients are heavy smokers or passive smokers, we quantified HMGB1 in these patients and compared the data with healthy smokers. Surprisingly, in our cohort of patients, a significantly low HMGB1 concentration compared with healthy smokers was detected.

#### Changes in Plasma HMGB1 Concentration During Follow-Up of Lung Adenocarcinoma Patients

In the cohort of patients with lung adenocarcinoma, no significant differences were observed in HMGB1 plasma concentration at t0, t1, or t2 (**Table 1** and **Figure 4B**). According to the Kaplan–Meier curve and based on median OS, the patients were divided into shorter-than-12-months (≤12) and longer-than-12-months (≥12) groups. Although no significant differences were observed, a tendency of increased HMGB1 plasma concentration in patients with longer OS was detected. According to **Table 1**, the plasma concentrations of HMGB1 at different time points as well as the other clinical parameters showed no significant differences between the groups with OS shorter than 12 months (≤12) or longer than 12 months (≥12) (**Figure 4B**).

#### Plasma HMGB1 Concentration in Patients From the LUAD-TCGA Project and Relationship to OS

Based on the data obtained from the LUAD-TCGA project, a survival analysis was performed. Two groups of patients were obtained according to HMGB1 expression, and OS was analyzed. According to the data, CDDP-treated patients expressing high levels of HMGB1 exhibited longer survival (**Figure 4C**).

#### Plasma HMGB1 Concentration in Lung Adenocarcinoma Patients and Relationship to OS

A tendency of increased plasma HMGB1 concentration was measured in patients with longer OS. To distinguish patients who responded to CDDP-based chemotherapy, a cutoff value for plasma HMGB1 concentration was calculated using ROC curves. Previously, it was observed that patients with survival longer than 12 months had higher plasma HMGB1 concentrations (**Figure 4B**). Therefore, survival curves were generated using a high cutoff value of 3.0 ng/mL (85% sensitivity and 23% specificity) for the three samples collected (**Table 5**). High HMGB1 plasma concentrations were significantly associated with patients having a longer survival (**Figures 4D–F**).

**Table 5:** Area under the receiver operating characteristics curve and optimal cutoff value of HMGB1.

	AUC	p-value	Sensitivity (%)	Specificity (%)	Cutoff^a^ (ng/mL)
t0	0.68	0.004	85.0	23.1	3.0
t1	0.67	0.008	82.3	23.1	3.0
t2	0.62	ns	76.9	23.1	3.0

AUC, area under curve.

a Cutoff value.

Because the design of our study was longitudinal and the time when plasma was collected is an important and dependent variable, an extended Cox model was constructed. Based on the above details, the following model was generated:

OS~[HMGB1] + age + gender + Karnofsky + ClusterID

Data obtained from this model are presented in **Table 6**. According to this model, the hazard ratio for plasma HMGB1 concentration indicated that its increase along with treatment is associated with longer OS. In conclusion, our data show that plasma HMGB1 concentration is directly correlated with patient survival.

**Table 6:** Extended Cox model data.

Variables	HR	RC	p-value	Confidence Interval of HR
HMGB1 (ng/mL)	0.717	1.39	0.004**	0.57–0.90 1.09–2.70 0.97–1.00 0.95–1.00
Gender	1.72	0.58	0.017*	
Age	0.990	1.00	0.328	
Karnofsky	0.970	1.02	0.067	

HMGB1, plasmatic concentration of HMGB1; HR, hazard ratio; RC, regression coefficients.

p-value Wald test = 0.02, concordance = 0.660. *p < 0.05, **p < 0.005.

## Discussion

In this study, we examined the role of HMGB1 in lung cancer cell lines and its biological importance during exposure to CDDP, which is the principal drug used in the treatment of lung cancer patients. Reports indicate that, during DNA damage repair mechanisms, HMGB1 plays an important role (7). In addition, HMGB1 acts as a DAMP associated with CDDP-induced cellular stress and ICD (31).

Clinical trials report that cancer patients become resistant to CDDP following the initial cycles of treatment. Tumors may exhibit intrinsic resistance because of cell cycle arrest or the induction of a dormant state.

Our results employing cell lines showed variable sensitivity to CDDP. This susceptibility was not associated to a particular mutation or mutational profile. Tomasini et al. reported that patients whose tumors coexpressed *KRAS* and *TP53* mutations were associated with a poor response to therapy (41). According to this report, the SKLU-1 cell line, which was used in this study, coexpressed *KRAS* and *TP53* mutations and exhibited high resistance to CDDP. However, the other mutations detected in the cell lines were not associated with resistance to CDDP. A possible explanation for this discrepancy is the use of a 2D *in vitro* assay. However, some reports suggest that the effect of CDDP is independent of the type of cell culture employed (42). Interestingly, we found that 1.3.11 cell line, harboring *PI3KCA*, *BRAF*, and *TP53* mutations, exhibited a high sensitivity to CDDP. Although whole-genome or whole-exome sequencing studies are needed, our results open the possibility to analyze these mutations in biopsies from lung cancer patients treated with platinum compounds and associate them with treatment response and OS.

Since the A549 cell line offers a better observation of the spatial–temporal morphological and biochemical changes associated with apoptosis and secondary necrosis following CDDP exposure, we analyzed this cell line for ICD markers. Following the recommendations from the committee on cell death (43), a sequential detection of calreticulin relocalization on the cell membrane and the release of ATP and HMGB1 were observed. An increase of HMGB1 was detected in cell culture supernatants during the process of secondary apoptosis/necrosis and corroborated by the release of LDH. These results suggest that, after exposure to CDDP, dying cells are the primary source of released HMGB1 in the supernatant.

As was previously reported, persistent cells are those that remain viable following the first exposure to cytotoxic drugs (6). Reports also indicate that HMGB1 plays an important role in the development of resistance to CDDP (15, 16). In this work, the possible contribution of HMGB1 to intrinsic resistance to platinum therapy, particularly in the persistent cells, was analyzed. HMGB1 shuttles between the cytoplasm and nuclei and performs distinct biological roles. Most of the untreated cells display HMGB1 localized in the cytoplasm. Persistent cells translocated HMGB1 to the nuclei. A possible explanation for this result is that nuclear HMGB1 in persistent cells may participate in the repair of CDDP-DNA lesions through NHEJ (9). However, more studies are required to confirm this hypothesis.

Reports indicate that HMGB1 binds to the extracellular receptors TLR-2, TLR-4, and RAGE. These receptors trigger signaling pathways to sustain inflammation and promote tumor development. In some cancer types, RAGE is expressed by malignant cells (44) and favors some hallmarks of cancer. In our study, CDDP-persistent cells over-expressed HMGB1 and RAGE, suggesting that the early activation of this feedback loop could be important for the induction of resistance to this cytotoxic drug (44). This suggestion is derived from the high fold-change of HMGB1 and RAGE expression observed in A549 and SKLU-1 cell lines, indicating that CDDP or soluble factors released by persistent cells could enhance their expression.

Cigarette smoke leads to a chronic inflammatory response associated with diverse pathologies. In healthy smokers, local and systemic inflammation have been reported (45). Because HMGB1 is an important player in inflammation, it is not surprising that we observed an increase in plasma HMGB1 in healthy smokers. In addition, smoking is an important risk factor in the development of lung cancer. Lower HMGB1 levels in patients compared with healthy smokers may result from HMGB1 binding to surface receptors expressed on tumor cells. In addition to the interaction with tumor cells, HMGB1 could bind to immune cells or other cell types in the tumor microenvironment to elicit a pro-tumoral activity. Previous reports indicate that tumor cells express RAGE, TLR-2, and TLR-4 to promote HMGB1-induced tumor growth.

The clinical relevance of HMGB1 as a biomarker associated with patient OS was studied. A preliminary analysis using data from the LUAD-TCGA project, curated using similar clinical criterion for those in our cohort, revealed that higher HMGB1 expression was associated with a better OS. However, the results using this database only consider information about patients prior to treatment. For a more rigorous description of HMGB1 as a biomarker associated with monitoring therapy, a longitudinal design collecting serial samples of lung cancer patients was employed. The clinical response of lung cancer patients to CDDP-based chemotherapy depends on individual variation and tumor heterogeneity. Although cell lines exhibit less heterogeneity compared with of tumor specimens, data from *in vitro* assays may support the results obtained from patients. Gathering the information obtained from cell lines with respect to HMGB1 release and translating this information to the tumor suggest that, in lung cancer patients, tumors consisting of a vast proportion of CDDP-susceptible cells may release HMGB1 as a consequence of cell death. In this setting, patients would show improved OS. This work was focused on the role of HMGB1 in lung adenocarcinoma and its relevance as a biomarker of response to therapy and OS. However, further studies are required to determine whether plasma HMGB1 levels are associated with tumor mass measurements (Response Evaluation Criteria in Solid Tumors) following treatment.

In conclusion, the lung adenocarcinoma cell lines exhibited varying sensitivity to CDDP. The sensitivity and resistance were independent of the mutational profile of several cancer driver genes. However, cell death occurring in sensitive cell lines was accompanied with the release of HMGB1. This work also demonstrates that, after a single CDDP exposure, a proportion of cells displayed resistance, in which HMGB1 was translocated to the nuclei. As such, HMGB1 may participate directly or indirectly in resistance to standard chemotherapy.

## Data Availability Statement

The datasets presented in this study can be found in online repositories. The names of the repository/repositories and accession number(s) can be found below: NCBI under BioProject: PRJNA770999 and the sequencing-read archive under accession SRP341619.

## Ethics Statement

The studies involving human participants were reviewed and approved by the Comite de Ciencia y Bioetica Instituto Nacional de Enfermedades Respiratorias “Ismael Cosio Villegas” Mexico City, Mexico. The patients/participants provided their written informed consent to participate in this study.

## Author Contributions

DA-C, RC-D, MP-M, and JL-G organized the entire manuscript, wrote the draft, and revised the last version of the manuscript. MG-V, RC-D, and MP-M performed assays in lung adenocarcinoma cell lines. RC-D, MP-M, MM-F, and SA-R sequenced and analyzed the DNA from lung adenocarcinoma cell lines. RC-D and AS-A performed confocal microscopy assays. DA-C, MG-V, and C-GG collected plasma samples, measured HMGB1, and analyzed data. JL-G designed and arranged the tables and figures. All authors contributed to the article and approved the submitted version.

## Conflict of Interest

The authors declare that the research was conducted in the absence of any commercial or financial relationships that could be construed as a potential conflict of interest.

## Publisher’s Note

All claims expressed in this article are solely those of the authors and do not necessarily represent those of their affiliated organizations, or those of the publisher, the editors and the reviewers. Any product that may be evaluated in this article, or claim that may be made by its manufacturer, is not guaranteed or endorsed by the publisher.

## Supplementary Material

Supplementary Figure 1 Protein expression level of receptor for advanced glycation end products (RAGE; 43 kDa) in cisplatin (CDDP)-persistent and untreated cells from A549 and SKLU-1 cell lines. Protein bands detected at around 40 kDa from cytoplasmic (C) or membrane (M) extracts are shown from control (CT) or CDDP (Tx)-treated cells. β-Actin (42 kDa) was employed as a constitutive expression control. THP-1 cell line was used as a constitutive control of RAGE expression.
