# Supplementary figures and images for: Role of HMGB1 in Cisplatin-Persistent Lung Adenocarcinoma Cell Lines
Source: Front Oncol. 2021 Dec 13;11:750677. doi: 10.3389/fonc.2021.750677 (PMC8710495; doi:10.3389/fonc.2021.750677)

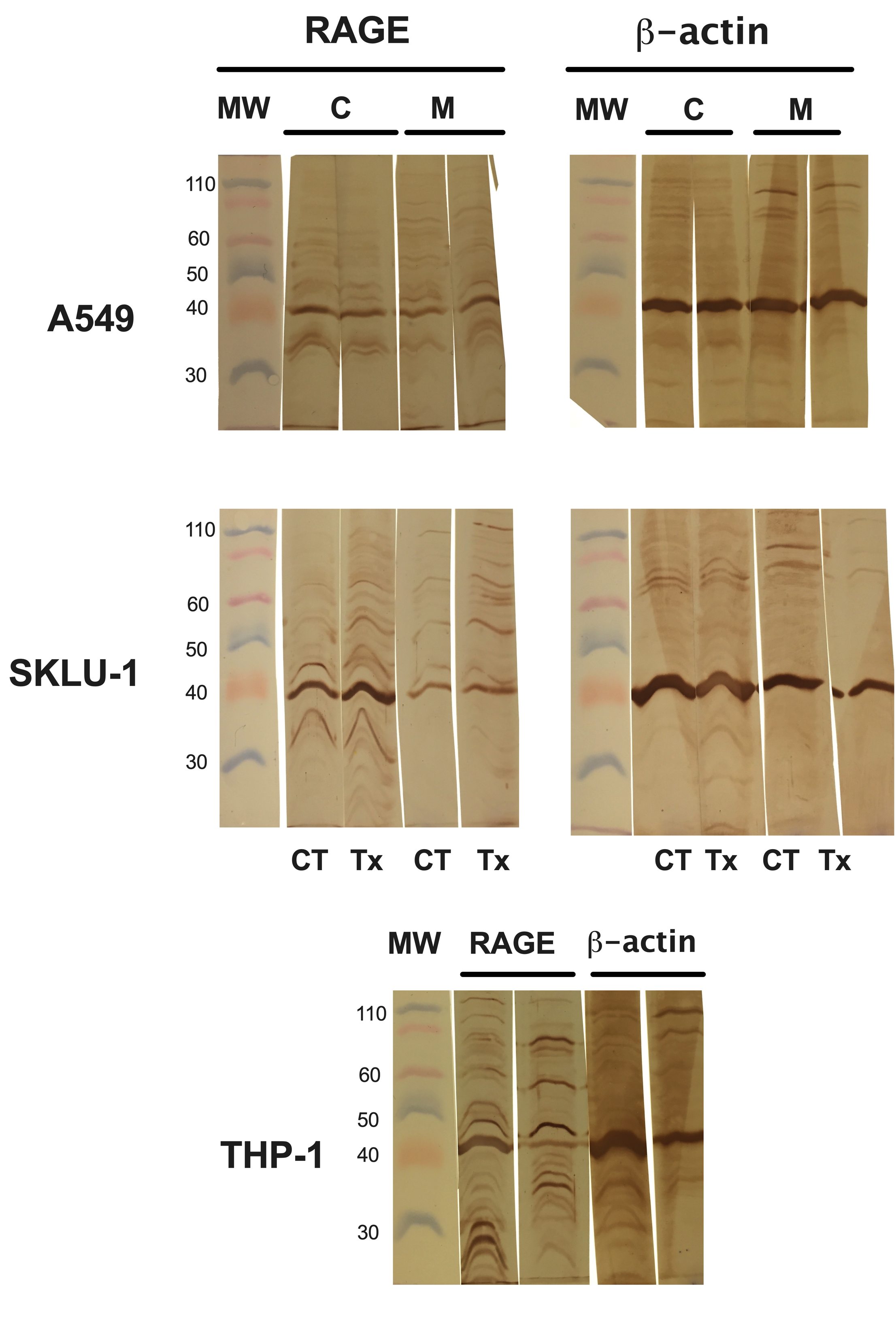

Supplement: Supplementary Figure 1 — Protein expression level of receptor for advanced glycation end products (RAGE; 43 kDa) in cisplatin (CDDP)-persistent and untreated cells from A549 and SKLU-1 cell lines. Protein bands detected at around 40 kDa from cytoplasmic (C) or membrane (M) extracts are shown from control (CT) or CDDP (Tx)-treated cells. β-Actin (42 kDa) was employed as a constitutive expression control. THP-1 cell line was used as a constitutive control of RAGE expression. [file Image_1.tiff]
